# Rethinking the role of clinical imaging

**DOI:** 10.7554/eLife.30563

**Published:** 2017-09-06

**Authors:** James PB O'Connor

**Affiliations:** 1 Division of Cancer SciencesUniversity of ManchesterManchesterUnited Kingdom; 2 The Christie HospitalManchesterUnited Kingdom

**Keywords:** radiomics, oncology, computational biology, imaging, genomics, biomarker, Human

## Abstract

Radiomics has the potential to improve the management of cancer patients, but further research is required before it can be adopted into routine clinical practice.

**Related research article** Grossmann P, Stringfield O, El-Hachem N, Bui MM, Rios Velazquez E, Parmar C, Leijenaar RTH, Haibe-Kains B, Lambin P, Gillies R, Aerts HJWL. 2017. Defining the biological basis of radiomic phenotypes in lung cancer. *eLife* **6**:e23421. doi: 10.7554/eLife.23421

The pressing need to improve cancer survival rates continuously motivates scientists to uncover the genetic and molecular composition of tumors, and use this information to develop more effective cancer therapies. Since tumors differ in their biological makeup, treatments can now often be tailored towards individual patients, in a strategy termed ‘personalized medicine’ (Schilsky, 2010). However, personalized medicine can only improve patient outcomes if there are reliable tests available to identify and analyze the biology of individual tumors, and detect which patients benefit from a given therapy (La Thangue and Kerr, 2011).

Medical imaging techniques underpin much decision-making in cancer medicine and can be used to determine, among other things, the size of a tumor and if it has spread into the surrounding tissue or other parts of the body. These and other tumor characteristics act as prognostic indicators and can help clinicians assign treatments for patients and monitor their response to therapy. However, only a few imaging techniques have been approved for use as companion diagnostic tools or as predictive biomarkers that can identify which patients are likely to benefit from a specific therapy (O'Connor et al., 2017). Now, in eLife, a team of researchers led by Hugo Aerts, Robert Gillies and Philippe Lambin – including Patrick Grossmann as first author – report on how an image analysis approach called radiomics could be used to enhance personalized medicine (Grossmann et al., 2017).

Radiomics uses computer algorithms to process the data collected by different medical imaging techniques and is becoming increasingly popular in cancer imaging research. One of its key characteristics is that radiomics recognizes that digital medical images are not only pictures – they are also are complex data. Radiomic analyses extract and measure an array of ‘features’ that describe the image texture and distributions of individual voxel values – the units that make up a 3D image – within a tumor. Each voxel represents a tiny amount of tissue and contains around 10^5^ to 10^7^ neoplastic and stromal cells, depending on tumor type and voxel dimensions.

Features can be descriptive terms derived from radiology reports or mathematical quantities. Feature extraction requires several complex steps in a defined pipeline: defining the regions of interest and segmenting three-dimensional images, extracting features and converting images into ‘mineable’ data. Finally, data from imaging techniques, and from biofluids and tumor tissue, are combined into models to predict patient prognosis or benefit from a specific therapy (Brady et al., 2016; Gillies et al., 2016).

Grossmann et al. – who are based at various insitiutes in the USA, Candada and The Netherlands – used radiomics to extract over 600 image features in computer tomography images from 262 patients with non-small cell lung cancer. They next examined how these features relate to the molecular characteristics of the tumors (such as the modulation of immune pathways and tumor suppressor proteins) and whether radiomic features predicted if the patients recovered after treatment. The findings were validated on an independent set of data from 89 other patients. Grossmann et al. discovered a connection between the radiomic phenotype of a tumor, the signaling pathways inside cells that drive how cancer develops, and clinical treatment outcomes. Moreover, by combining the data from radiomic, molecular and clinical studies, the overall chance of a patient surviving the cancer could be better predicted than when using clinical data alone.

The study by Grossmann et al. demonstrates the potential strength of radiomics, especially when combined with genomic and clinical information. One of the main benefits is that medical imaging data acquired in routine healthcare can be used in a new way to inform clinicians about the biology of a tumor and provide potential prognostic or predictive information. Imaging data from a large number of patients can be investigated rapidly. Further, radiomic image analysis is a non-invasive procedure that provides three-dimensional reconstructions of a tumor and avoids the problems associated with samples obtained through invasive biopsies (O'Connor et al., 2015).

However, several challenges need to be overcome before radiomics can be used as a companion diagnostic or predictive tool. Significant statistical problems can arise from analysis of such ‘big data’, with overfitting leading to relationships being seen were there are none (false discovery; Limkin et al., 2017). Once relationships are discovered, these findings need to be replicated in prospective studies.

Moreover, the terminology used for the derived image features needs to be standardized. At present, different investigators use their own names for the features, which makes it difficult to compare data between studies. In addition, the number of features incorporated into radiomic analyzes appears to vary even within individual research groups, leading to a phenomenon termed 'biomarker drift'. Biomarkers only become fit for guiding clinical decision-making when they become fixed in their acquisition, analysis and quality assurance processes, all defined clearly in standardized operating procedures (O'Connor et al., 2015).

Finally, ‘Picture Archiving and Communications System’ platforms must be adapted to integrate with radiomics algorithms in order to enable widespread clinical use. This will require coordinated efforts from clinical researchers and imaging scientists, along with additional computational power and infrastructure.

While considerable effort is required to rise to these challenges, the potential rewards are great. If enough evidence of the predictive power of radiomics can be produced, then radiomics may become a viable solution to help deliver personalized medicine in the clinic.
